# Optimization of preparation and transformation of protoplasts from *Populus simonii* × *P. nigra* leaves and subcellular localization of the major latex protein 328 (MLP328)

**DOI:** 10.1186/s13007-023-01128-5

**Published:** 2024-01-04

**Authors:** Ping Yang, Yao Sun, Xin Sun, Yao Li, Lei Wang

**Affiliations:** Department of Biotechnology, Institute of Advanced Technology, Heilongjiang Academy of Sciences, Harbin, 150001 China

**Keywords:** *Populus simonii × P. nigra*, Protoplast preparation, PEG-mediated transfection, Subcellular localization

## Abstract

**Background:**

*Populus simonii × P. nigra* is an ideal material for studying the molecular mechanisms of woody plants. In recent years, research on *Populus simonii* × *P. nigra* has increasingly focused on the application of transgenic technology to improve salt tolerance. However, the rapid characterization of gene functions has been hampered by the long growth cycle and exceedingly poor transformation efficiency. Protoplasts are an important tool for plant gene engineering, which can assist with challenging genetic transformation and the protracted growth cycle of *Populus simonii* × *P. nigra*. This study established an optimized system for the preparation and transformation of protoplasts from *Populus simonii* × *P. nigra* leaves, making genetic research on *Populus simonii* × *P. nigra* faster and more convenient. Major Latex Protein (MLP) family genes play a crucial role in plant salt stress response. In the previous study, we discovered that *PsnMLP328* can be induced by salt treatment, which suggested that this gene may be involved in response to salt stress. Protein localization is a suggestion for its function. Therefore, we conducted subcellular localization analysis using protoplasts of *Populus simonii* × *P. nigra* to study the function of the *PsnMLP328* gene preliminarily.

**Results:**

This study established an optimized system for the preparation and transformation of *Populus simonii* × *P. nigra* protoplasts. The research results indicate that the optimal separation scheme for the protoplasts of *Populus simonii* × *P. nigra* leaves included 2.5% cellulase R-10, 0.6% macerozyme R-10, 0.3% pectolyase Y-23, and 0.8 M mannitol. After enzymatic digestion for 5 h, the yield of obtained protoplasts could reach up to 2 × 10^7^ protoplasts/gFW, with a high viability of 98%. We carried out the subcellular localization analysis based on the optimized transient transformation system, and the results indicated that the MLP328 protein is localized in the nucleus and cytoplasm; thereby proving the effectiveness of the transformation system.

**Conclusion:**

In summary, this study successfully established an efficient system for preparing and transforming leaf protoplasts of *Populus simonii* × *P. nigra*, laying the foundation for future research on gene function and expression of *Populus simonii* × *P. nigra*.

**Supplementary Information:**

The online version contains supplementary material available at 10.1186/s13007-023-01128-5.

## Introduction

Protoplasts are the exposed cells that are enveloped by the plasma membrane after removing the cell wall through enzymatic or mechanical means [1]. Protoplasts are a promising tool that has been widely applied in various aspects of plant research, such as subcellular localization [2], protein interactions [3], cell fusion [4], and regenerated plants [5]. Enzymatic hydrolysis, which is simple and time-saving, is the most commonly used method for preparing protoplasts. However, suitable separation schemes need to be developed based on the different components in the cell walls of different species [6]. Factors such as enzyme concentration, osmotic pressure, and enzymolysis time can affect the yield and activity of protoplasts [7–9]. At present, protoplasts have been isolated from various plant tissues [10], with leaves being the most common [11]. The methods for separating leaf protoplasts have also been established in many plants, such as *Arabidopsis* [12], castor (*Ricinus communis* L.) [13], Areca palm (*Areca catechu*) [14], Eggplant (*Solanum melongena* L.) [15], *Cymbidium orchid* [16], and Perennial ryegrass (*Lolium perenne* L.) [17].

Transient gene expression in protoplasts has been widely used for subcellular localization assays. PEG-mediated protoplast transformation is one of the most commonly used methods [18]. In addition to low cost and simple operation, PEG-mediated transformation causes minimal damage to the protoplasts [19]. The conversion rate of transient expression mainly depends on plasmid concentration, PEG concentration, and transformation time [20, 21]. The protein subcellular localization is fundamental for understanding its biological functions at the cellular level. Relevant gene functions can be quickly and efficiently analyzed by optimizing the transient transformation system of protoplasts [22–24].

*Populus simonii* × *P. nigra* is an excellent variety cultivated through hybridization between *P. simonii* and *P. nigra*. It is widely distributed in the northern region of the Yellow River Basin in China and has a certain degree of stress resistance. It is an ideal material for studying the molecular mechanisms of woody plants. Using transgenic technology to improve salt tolerance has become one of the focuses of poplar research in recent years [25–27]. Many studies have shown that homologous genes of the Major Latex Protein (MLP) play a crucial role in plant salt stress response [28, 29]. In our previous study, we found that the Major Latex Protein PsnMLP328 can interact with the PsnERF76 transcription factor. We studied the expression of *PsnMLP328* in *Populus simonii* × *P. nigra* leaves under salt stress using Real-time PCR, and the results showed that the *PsnMLP328* gene can respond to salt stress [30]. Therefore, we want to preliminarily study the expression and function of the *PsnMLP328* gene in the protoplasts of *Populus simonii* × *P. nigra*. Currently, methods for protoplast preparation and transformation have been reported for *Populus euphratica* Oliv. [31], simon poplar (*Populus simonii*) [32], and hybrid poplar (*Populus nigra × P. maximowiczii*) [33], but reports on *Populus simonii* × *P. nigra* are limited. Therefore, it is necessary to establish an efficient system for protoplast preparation and transformation of *Populus simonii* × *P. nigra*.

This study established an effective method to optimize some factors that affect the protoplast preparation, such as enzyme concentration, mannitol concentration, and enzymolysis time. The factors affecting transformation, such as plasmid concentration, PEG4000 concentration, and transformation time, were optimized and subcellular localization of the PsnMLP328 was carried out using GFP as the reporter gene. This study lays the foundation for further research on the *PsnMLP328* gene, and also provides materials for future research on functional genes in *Populus simonii* × *P. nigra*.

## Materials and methods

### Plant material and growth conditions

This study used *Populus simonii* × *P. nigra* tissue culture seedlings from the tissue culture room of the Institute of Advanced Technology of Heilongjiang Academy of Sciences. The subcultured plantlets were cultured in a 400 mL tissue culture flask (high: 11 cm, diameter: 8 cm) using ½ MS medium (pH = 5.8) containing 20 g/L sucrose and 5 g/L agar. The plantlets were grown in an illumination incubator under a light/dark cycle with light intensity of 90 µmol m^−2^ s^−1^ at 26 °C for 16 h and darkness at 22 °C for 8 h for several months.

### Protoplast preparation and purification

The protoplast preparation and purification were partially modified based on Yoo and Huang’s protocol [34, 35]. Take the 2-4th young true leaves (0.2 g) from the top of the seedlings, remove the main veins of the leaves with a blade, and cut the leaves into thin strips of about 0.5–1 mm along the direction of the lateral veins. Spread the finely cut leaves with their paraxial surface facing downwards in a 90 mm culture dish containing 10 mL of enzymatic solution, and shake them under dark conditions at 27 °C, 80 rpm to promote the protoplast release. The composition of the enzymatic solution included 20 mM MES (pH = 5.8), 20 mM KCl, 0.1% BSA, 10 mM CaCl_2_, and different concentrations of cellulase R-10 (Yakult), macerozyme R-10 (Yakult), pectolyase Y-23 (Kyowa), and mannitol. To optimize the concentration of enzymes and mannitol, we first set four levels based on cellulase R-10, macerozyme R-10, and pectolyase Y-23 (Table 1). Therefore, an orthogonal experiment with three factors and four levels (L_16_(4^3^)) was used, with a total of 16 treatments; Mannitol concentration was set at four levels: 0.5 M, 0.6 M, 0.7 M, and 0.8 M. To optimize the enzymolysis time, the tissues were digested for 3, 4, 5, and 6 h, respectively.

**Table 1:** Orthogonal experimental factor level table

Level	Factor		
	A cellulase R-10 (%)	B macerozyme R-10 (%)	C pectolyase Y-23 (%)
1	1	0	0
2	1.5	0.2	0.1
3	2	0.4	0.3
4	2.5	0.6	0.5

After removing undigested leaf tissue by 38 μm (400 mesh) sieve filtration, collect the sample with a 50 mL centrifuge tube (29 × 104 mm, BECKMAN COULTER) and centrifuge at 100×*g* for 2 min at 4 °C. Carefully remove the supernatant and retain 2 mL of sediment. Add an equal volume of W5 solution (2 mM MES (pH = 5.8), 5 mM KCl, 125 mM CaCl_2_, 154 mM NaCl, and 5 mM glucose), wash the protoplasts twice at 4 °C, centrifuge at 100×*g* for 2 min, and then incubate on ice for 30 min. Wash the protoplasts again with MMG solution (4 mM MES (pH = 5.8), 0.8 M mannitol, and 15 mM MgCl_2_) and centrifuge at 4 °C for 2 min at 100×*g* to collect the protoplast precipitates. Observe the morphology of protoplasts under an optical microscope. After staining with 0.4% trypan blue, calculate the number of protoplasts (pieces/mL) and viability (%) using a cell counter. The protoplast yield was determined as follows:

$$\text{Protoplast}\; \text{yield}\; (\text{pieces}/\text{gFW}) = [\text{Number}\; \text{of}\; \text{protoplasts}\; (\text{pieces}/\text{mL}) \times \text{Protoplast}\; \text{volume}\; (\text{mL})]/{\text{total}}\; \text{leaf}\; \text{mass}\; (\text{gFW}).$$

### Plasmid construction

We chose the pBI121-GFP vector carrying the CaMV 35S promoter to characterize the transformation efficiency of the mesophyll protoplasts of *Populus simonii* × *P. nigra*. Plant total RNA was extracted from the leaves of Populus using the SDS method, and removal of the genomic DNA and reverse transcription was carried out using PrimeScript™ RT reagent Kit with gDNA Eraser (Takara, RR047A). *PsnMLP328* gene was amplified using specific primers (forward: 5′-GGCTAGAAGGAAATCAACG-3′; reverse: 5′-ATGAAGGCAAATGTATAGGTG-3′). After sequencing validation, specific primers (forward: 5′-CGCGCGGATCCATGGGCTAGCGGGAAAG-3′; reverse: 5′-TCCCCGGGGGCCTGACAAGGTTC-3′) were used to insert the target gene into the pBI121-GFP vector through the sites *Bam*HI and *Sma*I, resulting in a recombinant plasmid pBI121-MLP328-GFP. Plasmid Maxprep Kit (Vigorous) was used for plasmid extraction. Plasmid DNA was stored at − 20 °C, and its concentration was measured.

### Protoplast transformation

The improved PEG-mediated protoplast transformation method is based on Yoo and Huang’s protocol [34, 35]. The protoplast precipitates were resuspended to 10^5^ protoplasts/mL using an appropriate amount of MMG solution. 10 µL of different concentrations of pBI121-GFP plasmid DNA (20 µg, 40 µg, 60 µg, 80 µg) and 100 µL of protoplast suspension were added to a 2 mL tube and mixed gently, and then an equal volume of 110 µL PEG/Ca^2+^ solution (containing 100 mM CaCl_2_ and 0.8 M mannitol) was added and mixed gently. The concentration of PEG4000 in the PEG/Ca^2+^ solutions was set to 30%, 40%, 50%, and 60% for optimization. The 2 mL tubes were placed flat in the dark at room temperature for 10, 15, 20, and 30 min. 440 µL W5 solution (twice the volume of the incubation system) was added to the tube and mixed gently, and the supernatant was removed after centrifugation at room temperature for 2 min at 100×*g*. Add 500 µL of W5 solution and wash twice under the same centrifugal conditions. After resuspension with 1 mL of W5 solution, the tubes were placed flat in the dark at room temperature for 12–16 h. Centrifuge the tubes at 100×*g* for 2 min at room temperature, and then carefully remove the supernatant. The protoplasts expressing the green fluorescent protein (GFP) were observed and calculated with a confocal laser scanning microscope (LSM800, ZEISS) under the 10× objective microscopic field. The transformation efficiency was calculated using the following equation:

$$\text{Protoplast}\; \text{transformation}\; \text{efficiency}\; (\%) = (\text{number}\; \text{of}\; \text{fluorescent}\; \text{protoplasts}/{\text{total}}\; \text{number}\; \text{of}\; \text{protoplasts}) \times 100 \% .$$

### Subcellular localization

Based on the above optimized experimental protocols, the pBI121-MLP328-GFP fusion expression vector(as shown in “Plasmid construction” section) was transformed into the protoplasts of *Populus simonii* × *P. nigra* leaves. 10 µL of protoplast precipitates were collected by centrifugation and transferred to a glass slide. After covering the coverslip, the sample was inverted on the stage, and the fluorescence signals were detected by confocal laser scanning microscopy (LSM800, ZEISS) under the 20× objective microscopic field.

### Statistical analysis

All experiments were repeated three times. SPSS (27.0.1) was used for range analysis, and GraphPad Prism (8.3.0) was used for ANOVA and plotting.

## Results

### Preparation of protoplasts from *Populus simonii* × *P. nigra* leaves

**Fig. 1:**
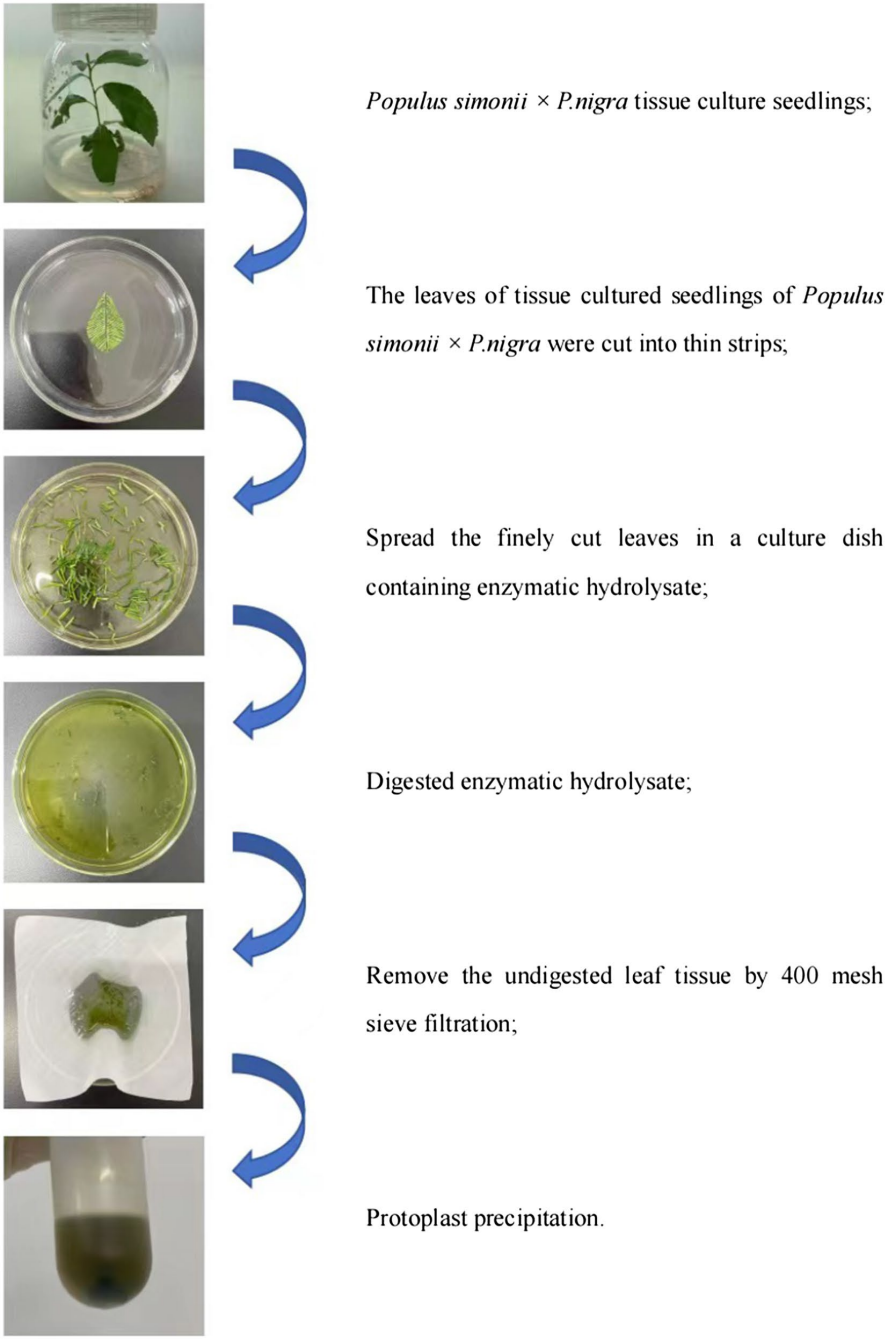
A brief overview for an efficient protocol for protoplast preparation from *Populus simonii* × *P. nigra* leaves

As shown in Fig. 1, a schematic protocol for protoplast preparation from *Populus simonii* × *P. nigra* leaves has been proposed. Enzyme combination, mannitol concentration, and enzymolysis time were optimized to establish a method for the preparation of protoplasts with high yield and viability.

**Fig. 2:**
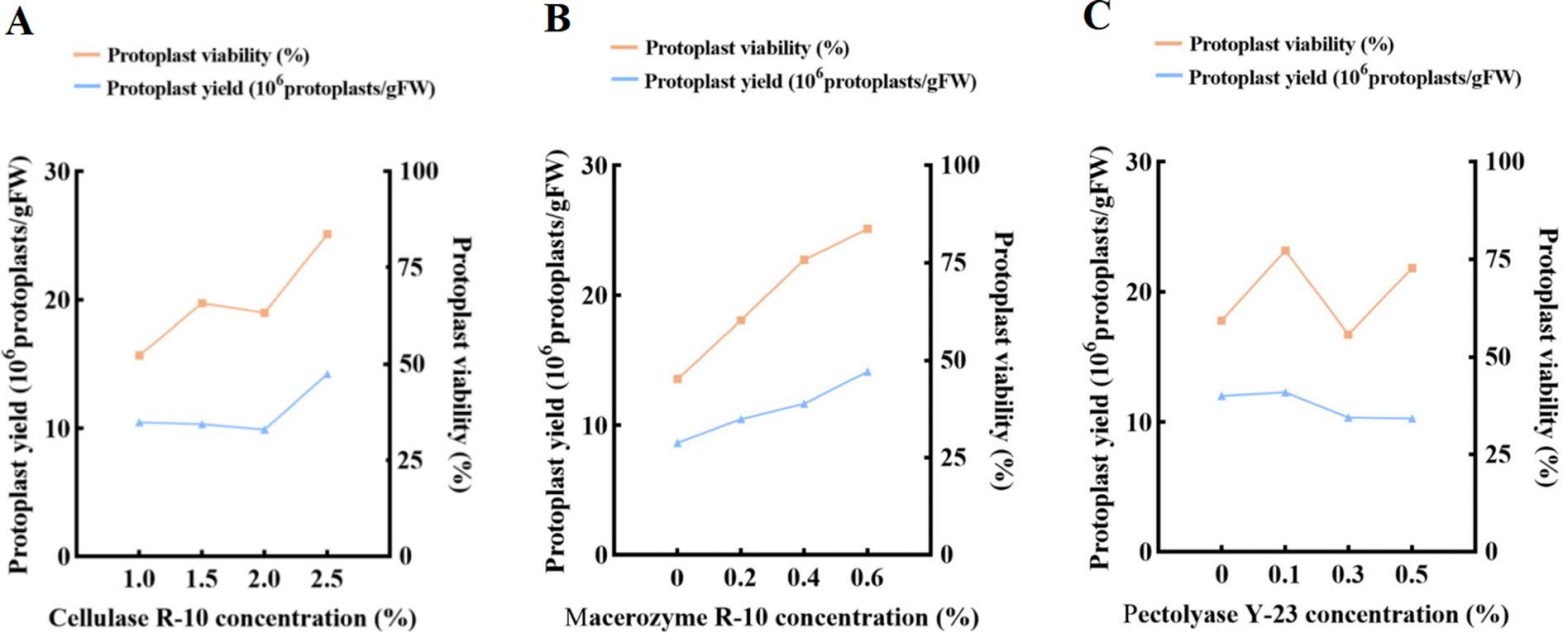
The effect of **A** cellulase R-10 concentration; **B** macerozyme R-10 concentration and **C** pectolyase Y-23 concentration on protoplast preparation from *Populus simonii* × *P. nigra* leaves

**Fig. 3:**
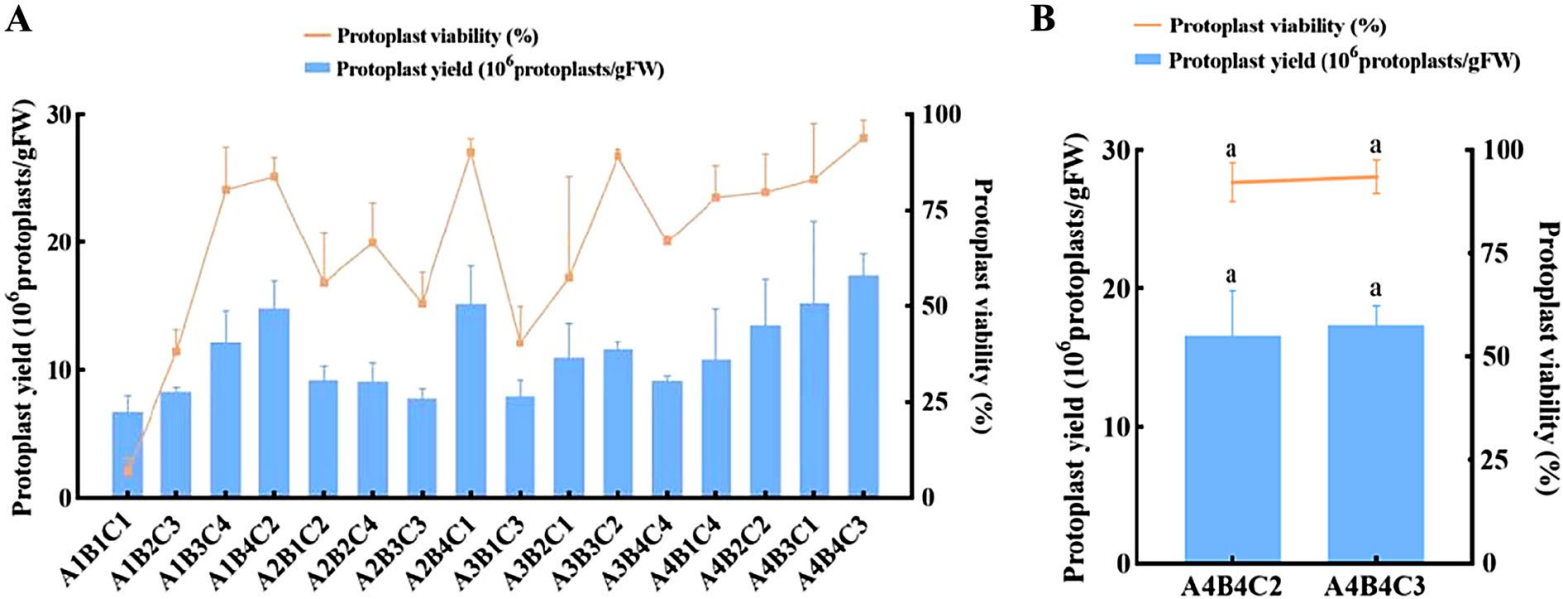
**A** Results of enzyme combination orthogonal experiment; **B** comparison between the predicted optimal enzyme combination and the actual optimal enzyme combination

We tested the effects of different concentrations of cellulase R-10, macerozyme R-10, and pectolyase Y-23 on protoplast yield and viability. The samples were placed in 0.6 M mannitol for 4 h. As shown in Fig. 2, with the increase of the concentration of macerozyme R-10, the yield and viability of protoplasts showed a similar upward trend. For cellulase R-10 and pectolyase Y-23, there were different trends between protoplast yield and viability with increasing enzyme concentration. The range analysis results of orthogonal experiments indicated that, macerozyme R-10, with the greatest impact on the yield and viability of protoplasts, was the most important factor, followed by cellulase R-10 and pectolyase Y-23 (Additional file 1: Table S1). It was indicated that the optimal combination was 2.5% cellulase R-10, 0.6% macerozyme R-10, and 0.1% pectinase Y-23, and both the yield and viability were optimal under the above enzyme concentration conditions. However, this enzyme concentration combination was not reflected in the orthogonal experiment. As shown in Fig. 3A, the yield and viability of protoplasts were maximal under the enzyme condition of 2.5% cellulase R-10, 0.6% macerozyme R-10, and 0.3% pectolyase Y-23 among all the treatments in the orthogonal experiment. Meanwhile, protoplast preparation was carried out based on the optimal combinations predicted by the orthogonal experiments and compared with the orthogonal experiments. When the concentration of pectolyase Y-23 was 0.3%, the yield of protoplasts was 1.74 × 10^7^ protoplasts/gFW and the viability was 94.0%, which was slightly higher than that of 0.1% pectolyase Y-23, but no significant difference was observed (Fig. 3B). In summary, the optimal enzyme concentration combination we have determined was 2.5% cellulase R-10 + 0.6% macerozyme R-10 + 0.3% pectolyase Y-23.

**Fig. 4:**
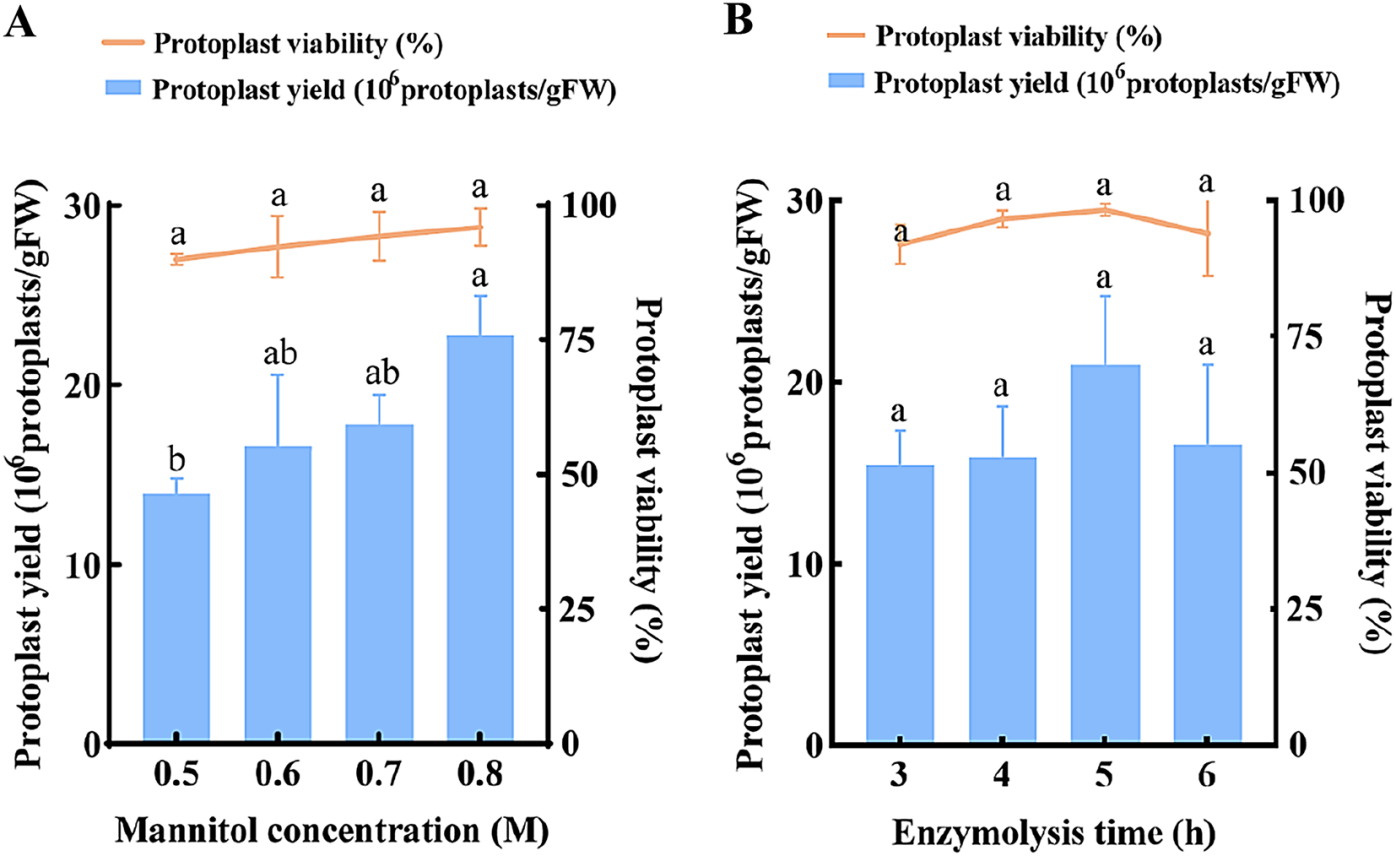
The effect of **A** mannitol concentration; **B** enzymolysis time on protoplast preparation from *Populus simonii* × *P. nigra* leaves

To determine the optimal osmotic pressure level, different concentrations of mannitol (0.5 M, 0.6 M, 0.7 M, and 0.8 M) were added to the optimized enzyme mixture, and the enzymolysis time was 4 h. The result showed an upward trend of the yield and viability of protoplasts with the increase of mannitol concentration. The protoplast yield (2.28 × 10^7^ protoplasts/gFW) and viability (96.0%) were significantly higher at 0.8 M mannitol than at 0.5 M, which both reached the maxima (Fig. 4A). In general, 0.8 M was determined as the optimal mannitol concentration.

To improve the final yield and viability of protoplasts, different enzymolysis times (3, 4, 5, and 6 h, respectively) were also analyzed with the enzyme solution containing 2.5% cellulase R-10 + 0.6% macerozyme R-10 + 0.3% pectolyase Y-23 and 0.8 M mannitol. As the enzymolysis time increased, the yield and viability of protoplasts first increased and then decreased. Although the effects of different enzymolysis times on the yield and viability of protoplasts were not significant, after 5 h of enzymatic hydrolysis, the highest protoplast yield (2.10 × 10^7^ protoplasts/gFW) was observed, and the viability (98.3%) was higher than other time points (Fig. 4B). In conclusion, 5 h was determined as the optimal enzymolysis time.

**Fig. 5:**
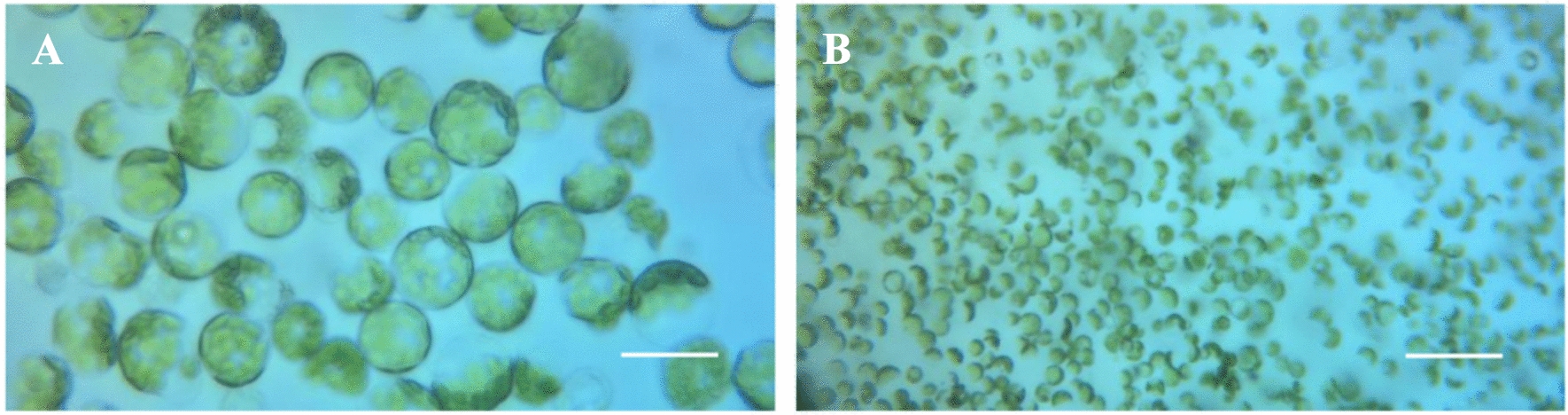
Protoplasts from *Populus simonii* × *P. nigra* leaves. **A** Bar = 25 μm; **B **bar = 100 μm

The protoplasts obtained by optimizing the conditions are full and intact in a circular shape, and the chloroplasts are clearly visible (Fig. 5).

### PEG-mediated transformation of *Populus simonii* × *P. nigra* mesophyll protoplasts

**Fig. 6:**
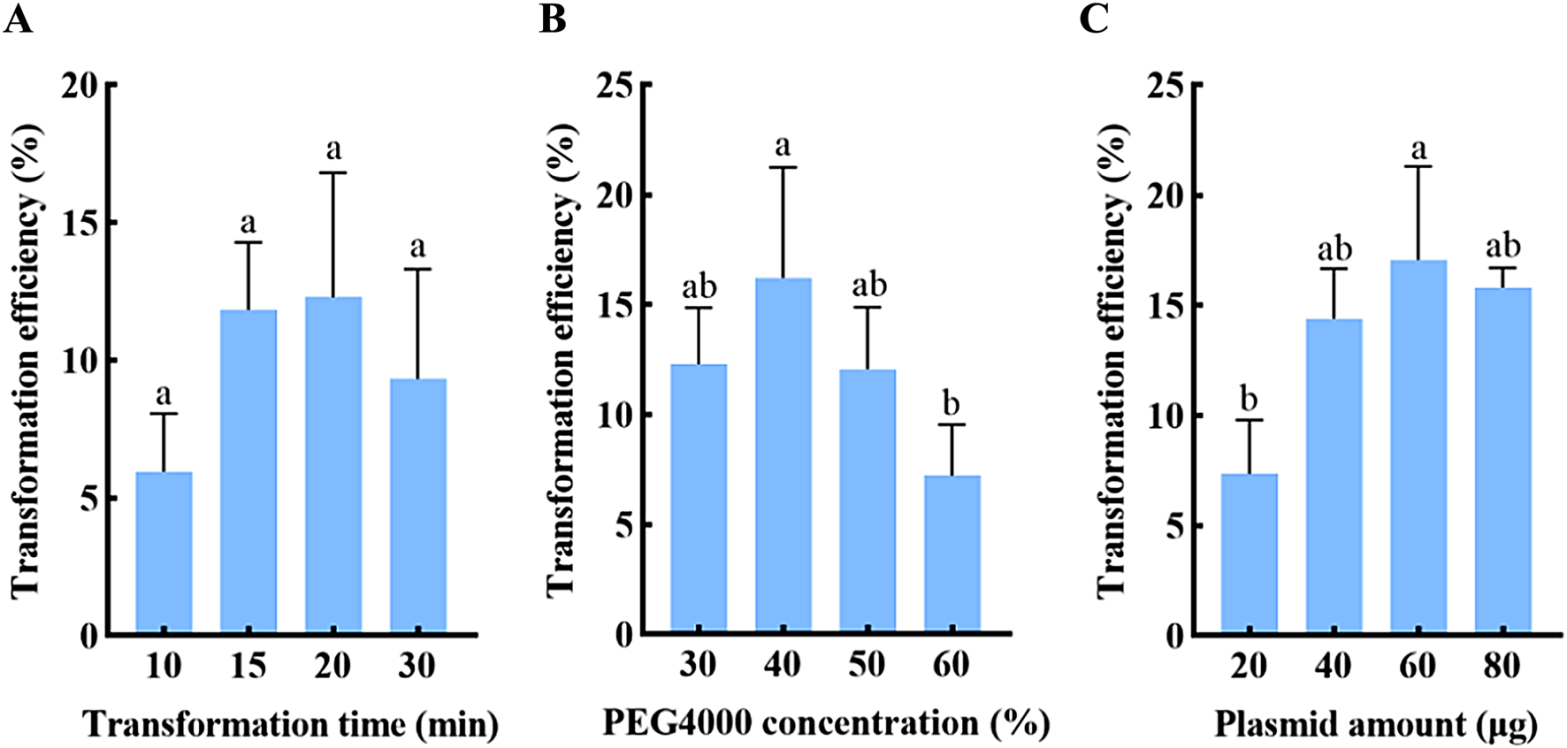
The effect of **A** transformation time, **B** PEG4000 concentration and **C** plasmid amount on protoplast transformation from *Populus simonii* × *P. nigra* leaves

In this study, PEG-mediated transient transformation of poplar protoplasts was adopted, and it was necessary to determine the effects of the amount of plasmid DNA, the concentration of PEG4000, and the transformation time on the transformation efficiency. To investigate the optimal transformation time, the samples were co-incubated with 20 µg plasmid and 40% PEG. The result suggested no significant difference in the transformation efficiency of protoplasts between different transformation times, and the transformation efficiency first increased and then decreased with the treatment time. When the transformation time was 20 min, the maximum transformation efficiency was 12.30% (Fig. 6A). To sum up, 20 min was determined as the optimal transformation time.

The effect of PEG4000 concentration on protoplast transformation efficiency was studied by incubation with 20 µg plasmid for 20 min. The result showed that the transformation rate increased and then decreased with the increase of plasmid concentration. When the PEG4000 concentration was 40%, the transformation efficiency reached its peak (16.23%), which is significantly higher than that of 60% PEG4000 (Fig. 6B). Therefore, 40% was selected as the optimal PEG4000 concentration.

The above transformation time and PEG4000 concentration were further applied to determine the proper plasmid amount. We observed that the transformation efficiency showed a trend of first increasing and then decreasing with the increased concentration. The protoplast transformation efficiency for 60 µg plasmid, which reached a maximum of 17.07%, was significantly higher than that of 20 µg (Fig. 6C). Overall, 60 µg was determined as the optimal plasmid amount. We also observed an upward trend of transformation efficiency from Fig. 6A–C, demonstrating that the overall transformation efficiency of the mesophyll protoplasts of *Populus simonii* × *P. nigra* was gradually enhanced by the optimization experiments.

### Subcellular localization analysis of MLP328 using *Populus simonii* × *P. nigra* mesophyll protoplasts

**Fig. 7:**
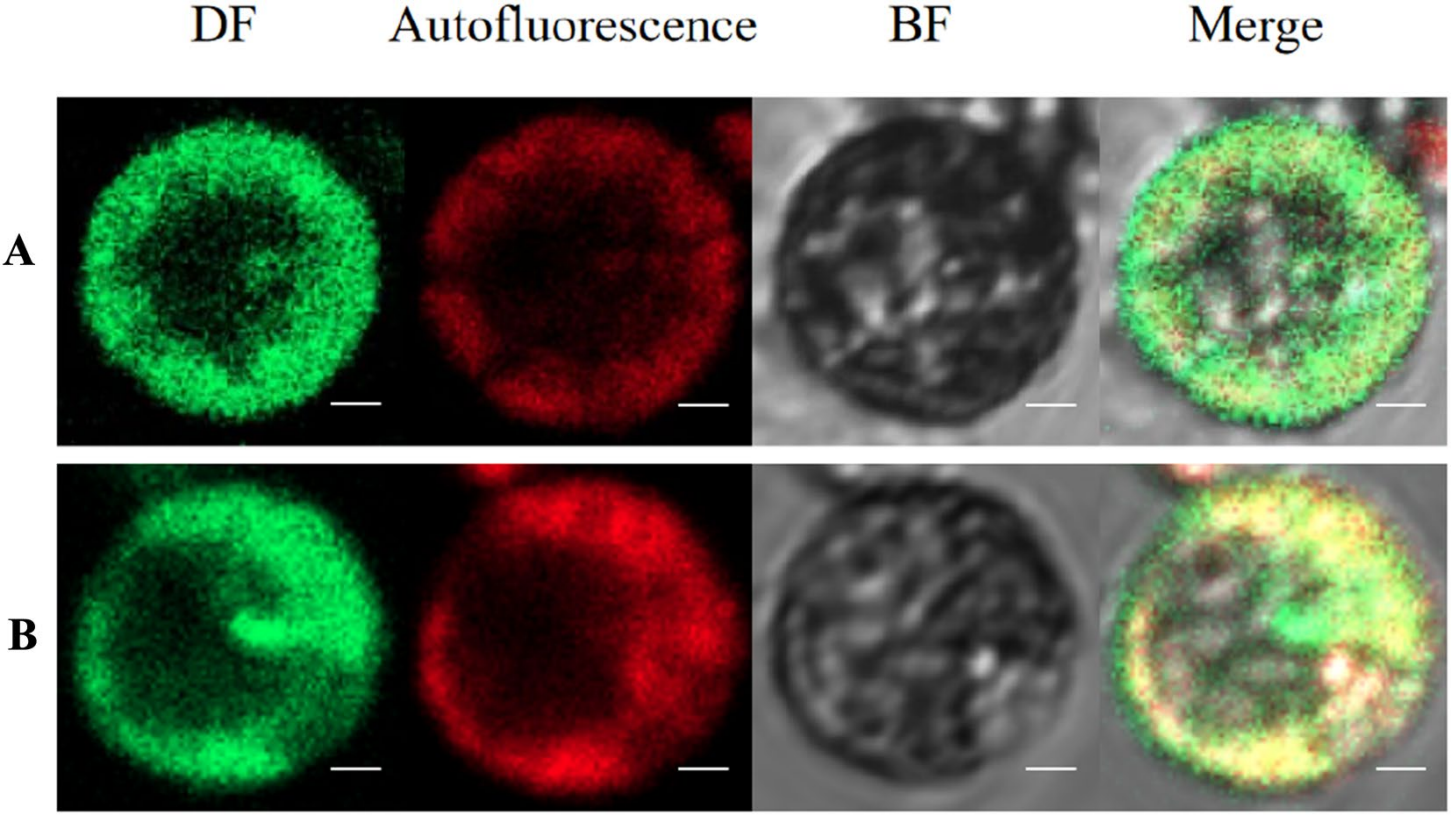
Subcellular localization of MLP328 protein in the protoplasts of *Populus simonii* × *P. nigra*. **A** pBI121-GFP empty vector; **B** pBI121-MLP328-GFP fusion protein expression vector. (*DF* dark field images, *autofluorescence* chloroplast autofluorescence images, *BF* bright field images, *merge* merged images, bar = 4 μm)

We used the above optimal experimental conditions to isolate the protoplasts, and the transient expression system was validated by examining the subcellular localization of PsnMLP328 in the leaf protoplasts of *Populus simonii* × *P. nigra*. The recombinant plasmid was successfully expressed in the protoplasts, indicating that MLP328-GFP signaling was specifically localized in the nucleus and cytoplasm. As a control, the pBI121-GFP empty vector showed GFP signaling in the nucleus, cytoplasm, and cell membrane (Fig. 7). Subcellular localization results proved the feasibility of the instantaneous expression system of protoplasts in the leaves of *Populus simonii* × *P. nigra*.

## Discussion

*Populus* is the main research genera in woody plants. To achieve rapid characterization of the gene function in populus, the transient transformation system of protoplasts is a reliable choice [10, 36]; therefore, it is necessary to establish a comprehensive preparation and transient transformation system of *Populus* protoplasts. The preparation of poplar protoplasts has been discussed in recent years. Guo et al. [31] obtained protoplasts from the suspension-cultured cell of *Populus euphratica* Oliv., and the highest protoplast yield (8 × 10^7^ protoplasts/gFW) with high viability (above 90%) was obtained using an optimized enzyme combination of 4% cellulase R-10 + 0.5% pectinase + 0.2% hemicellulase. They also optimized the PEG-mediated transformation system in which 20 µg plasmid DNA and 10^5^ protoplasts were co-incubated for 20 minutes, resulting in a transformation efficiency greater than 50%. Wang et al. [32] used the enzyme solution consisting of 0.5% Cellulase Onozuka RS, 1% Cellulase Onozuka R-10, 1% Macerozyme R-10, and 0.1% Pectolyase Y-23, enzymatic hydrolysis for 6 hours, and Simon poplar (*Populus simonii*) protoplasts were isolated from suspension cells, with protoplast yield of 3.8 × 10^7^ protoplasts/gFW. Park et al. [33] isolated protoplasts from leaf mesophyll of hybrid poplar (*Populus nigra × P. maximowiczii*) at a mean yield of 10.4 × 10^6^ protoplasts/gFW with mixture containing 0.8% Macerozyme R-10, 2.0% Cellulase ‘Onozuka’ R-10, 1.2% Hemicellulase, 2.0% Driselase, 0.05% Pectolyase Y-23, and 0.6 M mannitol. At present, there are relatively few examples of isolating protoplasts from poplar, and most of the protoplasts are derived from suspension cells. Many plant tissues and organs have been found to serve as materials for protoplast separation, such as roots [37], embryos [38], hypocotyls [39], suspension cells [31], stems [40], leaves [41], etc. Compared to other tissues, leaves are abundant, accessible, easy to operate, and suitable for quickly obtaining a large number of protoplasts, which makes it the most common material for protoplast isolation. The young leaves of tissue-cultured seedlings are ideal materials for protoplast isolation. Guo et al. [42] found it difficult to isolate protoplasts from *Populus* leaves grown in the field or greenhouse due to thick cuticles and recalcitrant cell walls. Therefore, this study used the top young leaves of tissue-cultured seedlings of *Populus simonii* × *P. nigra* as experimental materials.

This study established a system for the preparation and transformation of leaf protoplasts of *Populus simonii* × *P. nigra*. We first optimized the scheme for separating protoplasts from leaves of *Populus simonii* × *P. nigra*. Compositions of enzyme solution, osmotic pressure, and enzymolysis time have an impact on protoplast isolation [43]. Previous research have also described the different enzyme combinations used for protoplast isolation. For example, pectinase and cellulase are commonly used for leaf mesophyll protoplasts preparation, and have successfully obtained pea leaf protoplasts by optimizing conditions such as osmotic pressure, pH, and enzyme concentration [44]. Ma et al. [43] obtained a large number of spherical protoplasts from *C. bungei* with 3% Cellulase RS, 2% Macerozyme R-10, and 0.4 M mannitol in the enzymolysis solution, and the enzymolysis time was 8 h. Narasimhulu et al. [45] successfully isolated protoplasts from the hypocotyls of three genotypes of *Brassica carinata* seedlings after enzymatic hydrolysis with cellulase R-10 (0.5%) and pectolyase Y-23 (0.025%), using 0.4 M mannitol as an osmotic stabilizer. The dramatic variation in enzymes is due to the composition and structure of the cell walls of plant cells from different species and tissues, and the composition of the cell walls from the same species and organization may also differ at different stages. Therefore, it is necessary to develop the most suitable enzyme combination plan. This experiment investigated the use of cellulase R-10, macerozyme R-10, and pectolyase Y-23 to generate protoplasts under different enzyme concentration combinations using three factors and four levels (L_16_(4^3^)) orthogonal experimental design. It was predicted that the optimal enzyme concentration combination was 2.5% cellulase R-10 + 0.6% macerozyme R-10 + 0.1% pectolyase Y-23. However, this enzyme concentration combination was not reflected in the orthogonal experiment, and a simple superposition of each factor at the optimal level may not necessarily be the most suitable. Comparing the predicted combination with the optimal treatment in the orthogonal experiment, it was found that 2.5% cellulase R-10 + 0.6% macerozyme R-10 + 0.3% pectolyase Y-23 provided the higher protoplast yield and viability (Fig. 3B). It should be noted that both the maximum yield and viability of protoplasts were simultaneously achieved under the optimal enzyme combination, which may have a favorable impact on the subsequent experiments. Besides, it could also be inferred that macerozyme R-10 was the most important factor among the enzyme combination, which we believed might be closely related to the degradation of cell wall components and single-cell separation in *Populus simonii* × *P. nigra*. Pectin is widely present in tissues such as stems, leaves, and fruits of higher plants, and is an important component of plant intercellular matrix, serving as a cell adhesive. Macerozyme R-10 is a multi-enzyme mixture derived from *Rhizopus* sp. that possesses high pectinase and hemicellulase and low cellulase activity. The macerozyme R-10, as a pectin-degrading enzyme, is commonly used to break down the pectin in plant tissues, causing the cells bound together to dissociate into living individual cells. A suitable concentration of the macerozyme R-10 may significantly enhance the efficiency to degrade the pectin and isolate single cells in *Populus simonii* × *P. nigra* [46–49].

Stable osmotic pressure can maintain the balance between the internal and external environment of protoplasts, which is crucial for maintaining the morphology and state of protoplasts. Mannitol, sucrose, sorbitol, and glucose, are commonly used osmotic stabilizers [35]. Among them, mannitol is the most commonly used in protoplast separation [50]. In this experiment, we found that when the concentration of mannitol was 0.8 M, the yield and viability of protoplasts reached their maximum, and lower concentrations could cause protoplast fragmentation.

The enzymolysis time required for different plant materials varies. During the process of sugarcane protoplast preparation, Wu et al. [51] showed a trend of first increasing and then decreasing protoplast yield and viability with increasing enzymolysis time. Adjei et al. [52] found that the optimal enzymolysis time for mango protoplasts was 12 h, while Wang et al. [53] used 2 h for separation of passion fruit protoplasts. In this experiment, the enzymatic hydrolysis of plant tissue was observed every half hour to ensure that the protoplasts were not damaged or insufficiently digested due to enzymolysis time. The optimal enzymolysis time for leaves of *Populus simonii* × *P. nigra* is 5 h.

The key factors affecting protoplast transformation are plasmid concentration, PEG4000 concentration, and transformation time. Li et al. [54] indicated that transformation efficiency of 41.7% was achieved with 20% PEG-4000, 20 µg plasmid DNA, 2 × 10^5^ protoplasts/mL, and a transfection duration of 30 min. The parameters of the transient transformation system set by Du et al. [55] were as follows: the concentration of protoplasts is 5 × 10^5^ cells/mL, exogenous DNA concentration of 500 µg/mL, PEG4000 final concentration of 40%, and transformation time of 15 min. Han et al. [56] demonstrated that when the concentration of PEG4000 was 30–40%, the transformation efficiency of *Ginkgo biloba* protoplasts reached 40%. We successfully studied the localization of PsnMLP328 protein in the nucleus and cytoplasm by optimizing these factors and validated the feasibility of the instantaneous transformation system of the protoplasts of *Populus simonii* × *P. nigra*.

## Conclusion

This study established an efficient and reliable system for the preparation and transient expression of leaf protoplasts of *Populus simonii* × *P. nigra*. The optimized protocols provide technical support for further research on functional genomes and gene editing in the protoplasts of *Populus simonii* × *P. nigra*. Subcellular localization assay confirmed that the PsnMLP328 protein was located in the nucleus and cytoplasm, laying a preliminary research foundation for the subsequent study of the function and molecular mechanism of the *PsnMLP328* gene. In summary, the optimized method in this study not only provides materials for related research on *Populus simonii* × *P. nigra*, but also provides reference for the preparation and transformation of leaf protoplasts of other species.

### Supplementary Information

**Additional file 1: Table S1. **Orthogonal experiment of three enzymes affecting of protoplast isolation.

## Data Availability

The datasets supporting the conclusions of this article are included in the article.
