# Supplementary material for: Optimization of preparation and transformation of protoplasts from Populus simonii × P. nigra leaves and subcellular localization of the major latex protein 328 (MLP328)
Source: Plant Methods. 2024 Jan 4;20:3. doi: 10.1186/s13007-023-01128-5 (PMC10765669; doi:10.1186/s13007-023-01128-5)
Supplement: Supplementary file 1 — Additional file 1: Table S1. Orthogonal experiment of three enzymes affecting of protoplast isolation. [file 13007_2023_1128_MOESM1_ESM.docx]

**Table S1** Orthogonal experiment of three enzymes affecting of protoplast isolation

| Treatment combination | factor | | | Protoplast yield  (10^6^ protoplasts/gFW) | Protoplast viability  (%) |
| --- | --- | --- | --- | --- | --- |
|  | A cellulase R-10 (%) | B macerozyme R-10 (%) | C pectolyase Y-23 (%) |  |  |
| A1B1C1 | 1 | 0 | 0 | 6.72±1.05 | 7±2.87 |
| A1B2C3 | 1 | 0.2 | 0.3 | 8.28±0.29 | 38±4.90 |
| A1B3C4 | 1 | 0.4 | 0.5 | 12.11±2.06 | 80±8.96 |
| A1B4C2 | 1 | 0.6 | 0.1 | 14.83±1.74 | 84±4.03 |
| A2B1C2 | 1.5 | 0 | 0.1 | 9.18±0.90 | 56±10 |
| A2B2C4 | 1.5 | 0.2 | 0.5 | 9.10±1.17 | 66±8.65 |
| A2B3C3 | 1.5 | 0.4 | 0.3 | 7.78±0.61 | 51±6.60 |
| A2B4C1 | 1.5 | 0.6 | 0 | 15.18±2.41 | 90±2.83 |
| A3B1C3 | 2 | 0 | 0.3 | 7.93±1.04 | 40±7.85 |
| A3B2C1 | 2 | 0.2 | 0 | 10.93±2.24 | 57±21.56 |
| A3B3C2 | 2 | 0.4 | 0.1 | 11.56±0.52 | 89±1.41 |
| A3B4C4 | 2 | 0.6 | 0.5 | 9.13±0.33 | 67±1.25 |
| A4B1C4 | 2.5 | 0 | 0.5 | 10.78±3.28 | 78±6.65 |
| A4B2C2 | 2.5 | 0.2 | 0.1 | 13.53±2.92 | 80±8.01 |
| A4B3C1 | 2.5 | 0.4 | 0 | 15.24±5.24 | 83±12.03 |
| A4B4C3 | 2.5 | 0.6 | 0.3 | 17.39±1.37 | 94±4.03 |
|  | K1 | 41.94 | 34.61 | 48.07 |  |
|  | K2 | 41.24 | 41.84 | 49.1 |  |
|  | K3 | 39.55 | 46.69 | 41.38 |  |
|  | K4 | 56.94 | 56.53 | 41.12 |  |
|  | k1 | 10.48 | 8.65 | 12.02 |  |
| Protoplast yield | k2 | 10.31 | 10.46 | 12.28 |  |
|  | k3 | 9.89 | 11.67 | 10.34 |  |
|  | k4 | 14.23 | 14.13 | 10.28 |  |
|  | Range | 4.35 | 5.48 | 1.99 |  |
|  | Rank | B>A>C |  |  |  |
|  | **Optimal combination** | A4B4C2 |  |  |  |
|  | K1 | 209 | 181 | 237 |  |
|  | K2 | 263 | 241 | 309 |  |
|  | K3 | 253 | 303 | 223 |  |
|  | K4 | 335 | 335 | 291 |  |
|  | k1 | 52.25 | 45.25 | 59.25 |  |
| Protoplast viability | k2 | 65.75 | 60.25 | 77.25 |  |
|  | k3 | 63.25 | 75.75 | 55.75 |  |
|  | k4 | 83.75 | 83.75 | 72.75 |  |
|  | Range | 31.5 | 38.5 | 21.5 |  |
|  | Rank | B>A>C |  |  |  |
|  | **Optimal combination** | A4B4C2 |  |  |  |
